# Peri-operative stroke: isolated oculomotor palsy following Hartmann's procedure

**DOI:** 10.1097/EA9.0000000000000045

**Published:** 2024-01-25

**Authors:** Craig Alistair Wood

**Affiliations:** From the Department of Critical Care, Northumbria Specialist Emergency Care Hospital, Northumbria Way, Cramlington, Northumberland, UK (CAW)

## Introduction

Peri-operative stroke can be defined as any haemorrhagic or ischaemic cerebrovascular accident that occurs within 30 days of the initial surgery. Such strokes are frequently thromboembolic in origin and commonly occur within the first 72 postoperative hours after a symptom-free interval.^1^ Multiple factors contribute to their pathophysiology, including patient-related and environmental factors. The incidence rate can also be influenced by the type and complexity of surgery performed. To date, research has supported a causal relationship between hypotension and peri-operative stroke. This highlights blood pressure management as being critical during the intra-operative period. However, the extent to which hypotension contributes to peri-operative stroke remains unclear. All peri-operative strokes deserve significant attention because of their serious disability and high mortality. This ultimately leads to prolonged admissions and a potential need for long-term care.

## Case report

A woman in her 60s with a known diverticular sigmoid stricture presented with a 2-week history of abdominal pain and vomiting. She had a history of hypertension, type 2 diabetes (controlled with metformin), osteoarthritis, gastritis and diverticulitis. Computed tomography (CT) of the abdomen revealed large bowel obstruction secondary to stricture. Therefore, she underwent a Hartmann's procedure via laparotomy with a National Emergency Laparotomy Audit (NELA) score of 1.67% preoperatively.

Preoperative blood pressure was 197/81 mmHg [mean arterial pressure (MAP) 120 mmHg] and the initial intra-operative MAP target was to remain within 20 to 25% of baseline. Prior to induction, a spinal anaesthetic consisting of morphine sulphate (200 μg) in 2 ml of normal saline was administered preoperatively, according to local protocol. The patient was then anaesthetised using intravenous anaesthesia with concentration objective (AIVOC) using Remifentanil and Propofol. Rocuronium was used as a nondepolarising neuromuscular blocker to facilitate tracheal intubation. An arterial line was then sited for accurate haemodynamic monitoring with a urinary catheter to monitor urine output as a surrogate for renal perfusion.

Throughout the intra-operative period, blood pressure was labile, with a SBP ranging from 85 to 190 mmHg. During a period of significant intra-operative hypertension (systolic >170 mmHg), 3 g of intravenous magnesium was administered, which reduced the blood pressure to a good effect. Phenylephrine was then used to support blood pressure when the patient was hypotensive, and was frequently titrated or stopped to maintain a mean arterial pressure (MAP) greater than 65 mmHg. In total, 3146 mg of phenylephrine was administered intra-operatively because of significant periods of hypotension. Three litres of Lactate Ringers solution were also administered intra-operatively because of concerns that the patient was potentially hypovolaemic and septic given the increasing amounts of vasopressor required.

The total duration of surgery was 4 h 20 min and the duration of time in the postoperative recovery area was 2 h and 25 min. The patient was extubated prior to transfer to the recovery area and was maintained on 2 l of oxygen via nasal cannula. During the peri-operative period it was recorded that:

(1) Systolic less than 100 mmHg = 50 mins duration intra-operatively and 30 min in recovery
(2) MAP less than 80 mmHg = 110 min intraoperatively and 55 min in recovery
(3) MAP less than 70 mmHg = 30 min intraoperatively and 20 min in recovery
(4) MAP less than 60 mmHg = 10 min in recovery

As increasing amounts of phenylephrine were required in the recovery area, a decision was made to add noradrenaline because of its mixed alpha-1 and beta activity. This was administered using a central venous catheter and allowed the phenylephrine to be tapered down until the MAP was maintained solely on 6 mg min^−1^ of noradrenaline. Given the ongoing noradrenaline requirement, the patient was transferred across to the critical care unit to facilitate ongoing care.

Eleven hours after surgery during the morning critical care review, the patient was identified as having complete right ptosis with an inability to elevate her eyelid. Further examination revealed that her right eye had deviated downward and outward with a dilated right pupil. There was an inability to adduct and elevate the eye, with limitations in depressing the affected eye. This resulted in diplopia in the patient. She maintained some ability to abduct, thus confirming a functional trochlear and abducens nerve function.

She had an enlarged right pupil (5 mm) with a sluggish direct light reflex, compared with the left (3 mm). However, she maintained full visual fields, had no visual inattention and reported no other motor or sensory loss. Power in upper and lower limbs was 5/5, and neurological examination revealed no other cranial nerve deficit, including no evidence of speech disturbance. Glasgow coma scale at the time was 15/15 and haemodynamic observations were within normal limits [blood pressure 119/59 mmHg (MAP 78 mmHg), heart rate regular at 85 bpm, temperature 36.4 °C, respiratory rate 12 breaths minute^−1^, SpO_2_ 98% on room air, blood glucose 7.5 mmol l^−1^). The patient was, therefore, diagnosed with an isolated third-nerve palsy.

**Fig. 1:**
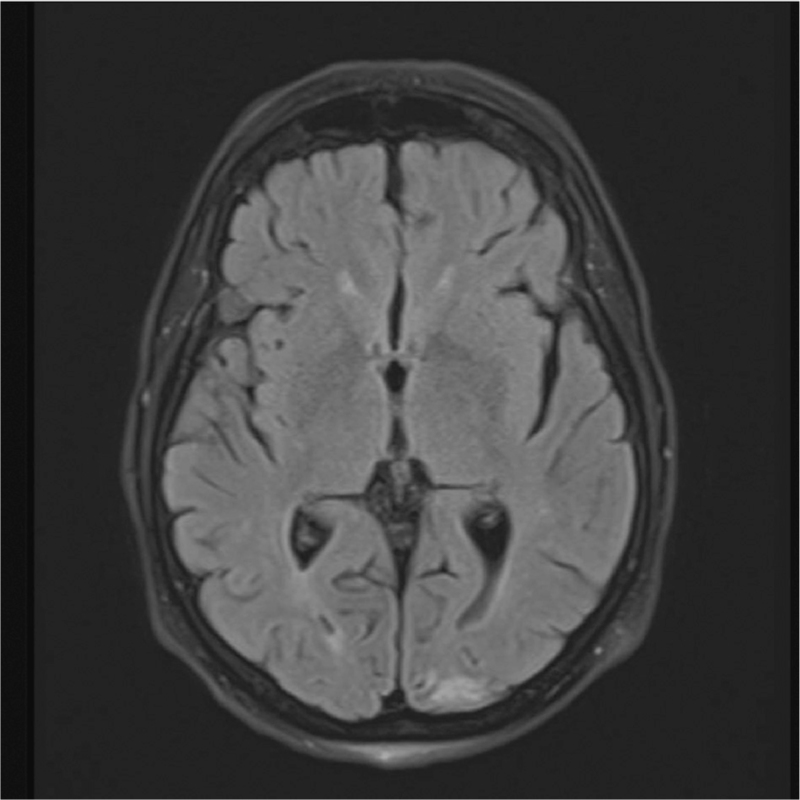
Axial FLAIR MRI showing diffusion restriction in left occipital lobe.

**Fig. 2:**
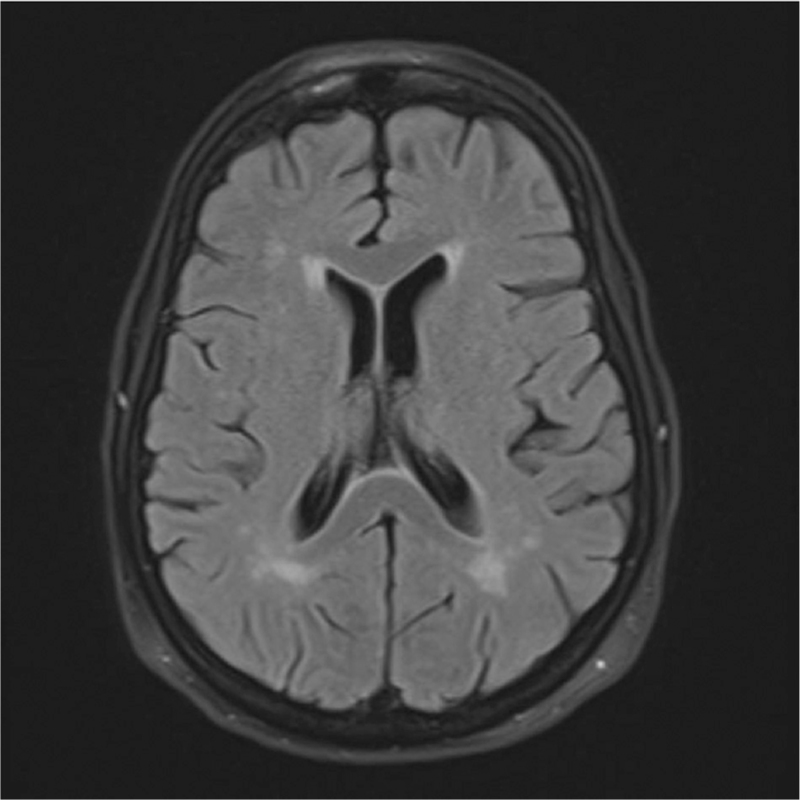
Axial FLAIR MRI acute infarcts bilaterally across corona radiata.

Given that metal surgical clips were *in situ*, the initial imaging was a head CT to rule out a haemorrhagic cause. This showed no acute findings; therefore, magnetic resonance angiography (MRA) was performed to rule out an intracranial aneurysm, particularly a posterior communicating aneurysm. The metal clips were removed from the midline incision as these are contra-indicated for MRA scans and were replaced with steri-strips by the surgical team. The MRA showed a normal Circle of Willis with no evidence of arterial stenosis or aneurysm. However, diffusion restriction was observed in the left occipital lobe, suggesting an acute infarct (Fig. 1). Tiny acute infarcts were also seen across the centrum semiovale and corona radiata (Fig. 2), which are thought to be embolic in nature. Therefore, the oculomotor palsy was likely due to microvascular ischaemia.

As there was no evidence of proximal large vessel occlusion, intra-arterial thrombolysis and mechanical clot retrieval could not be utilised. For ongoing management and on discussion with the stroke team, we aimed for Hb greater than 90 g l^−1^, SBP less than 180 mmHg, and prescribed aspirin 75 mg once daily as secondary prevention. Low-molecular-weight heparin (LMWH) was administered in the background. Further imaging, including transthoracic echocardiography, was performed to rule out a cardiac cause of the emboli. However, there was no evidence of vegetation or cardiac thrombus. She also underwent an ultrasound Doppler of both carotid arteries, which revealed reduced velocities in the left carotid artery, suggesting 50% stenosis. There was no evidence of paroxysmal atrial fibrillation during the operation, and the patient remained on continuous monitoring throughout her critical care admission.

On discharge, follow-up care was arranged with the neuro-ophthalmology and stroke team. Her aspirin was switched to clopidogrel 75 mg once daily, and she continued her LMWH, completing a 28 day course. She regained some ability to open her right eyelid on discharge 14 days later and was scheduled for ambulatory care appointments as an outpatient because of electrolyte disturbances. During these appointments, she voiced issues with depth perception, which affected her ability to manage her stoma. However, the partial ptosis reduced her double vision, serving as a natural protective factor. The stroke team advised a 72 h Holter tape to rule out paroxysmal atrial fibrillation, and she was discharged from their service with no intervention required for the 50% left carotid artery stenosis.

Neuro-ophthalmology reviewed her 4 weeks as an outpatient following the stroke event. She still experienced right ptosis with binocular diplopia but had no pupil involvement. She had normal visual fields with visual acuity of 6/6 right eye using pinhole and 6/6 left eye using pinhole. Ishihara colour vision was 13/13 in both eyes. A further neuro-ophthalmology was scheduled 8 weeks later. At this appointment, she voiced that her right ptosis had resolved (at 10 weeks post stroke) and her diplopia had resolved (at 11 weeks post stroke). In this appointment, visual acuity of her right eye was 6/6 unaided and her left eye was 6/9 with pinhole. She was, therefore, discharged from clinic with the right third cranial nerve palsy having resolved.

## Oculomotor palsies

A third cranial nerve palsy (oculomotor) commonly presents with ptosis, restricted ocular movements, exotropia, and diplopia. Mydriasis can also be present, depending on the site of the lesion, resulting in a dilated pupil that fails to constrict to light. Such palsies can be characterised as partial or complete. Complete closure indicates full eyelid closure, with muscles innervated by the third cranial nerve being severely affected. Sparing of the pupil can occur in complete palsy and would suggest that a compressive lesion (*i.e.* an aneurysm) is less likely.^2^ This conveys a strong anatomical link between the location of damage along the path of the nerve and the resulting features.

The various causes of oculomotor palsy include microvascular ischaemia, aneurysm, trauma, neoplasm, inflammation, or neurosurgical intervention.^3^ When no apparent cause can be identified, CT angiography or MRA is essential to rule out compression from an intracranial aneurysm. These aneurysms commonly arise from the posterior communicating artery and are associated with extremely high morbidity and mortality rates. Approximately 15% of patients with this aneurysm die before hospital admission, 40% die in hospital, and a third of survivors have permanent neurological deficits.^2^

As the patient's head CT and MRA showed no evidence of an aneurysm, trauma, or neoplasm, the underlying cause was likely microvascular ischaemia. This is often the result of injury to the very small blood vessels that supply the third cranial nerve. These blood vessels are too small to be viewed using neuroimaging techniques and too small to be affected by an embolus.

Microvascular ischaemia remains the most common cause of third nerve palsy and is mainly observed in hypertensive and diabetic patients. This typically presents as a complete, pupil sparing third nerve palsy, although up to 38% of cases are recorded to have some pupil involvement.^2^ Pain may or may not be present behind the eye, but it is an unreliable indicator for differentiating the cause. This is because traumatic, aneurysmal and arteritic third nerve palsies can also present with pain.

## Discussion

Peri-operative stroke is a relatively rare complication of surgery that can have devastating outcomes. When they occur, they are typically ischaemic rather than haemorrhagic and can be categorised as embolic, thrombotic or haemodynamic (hypoperfusion).^4^ The causes of peri-operative ischaemic stroke include atrial fibrillation, lacunar stroke, cerebral atherothrombosis, hypotension, dehydration and systemic hypercoagulability.^5^ In relation to our patient and her prolonged surgery, it is clear that intra-operative systemic hypercoagulability and hypotension were likely precipitants of the stroke. However, there were other contributing factors.

Hypertension and diabetes are strong vascular risk factors for strokes. Other risk factors for stroke include age older than 70 years, female sex, smoking, renal insufficiency, carotid stenosis, previous stroke, chronic obstructive pulmonary disease, peripheral vascular disease, cardiac disease including atrial fibrillation and discontinuation of antithrombotic therapy before surgery. Undergoing a surgical procedure also confers its own risk factors, including the timing of the procedure (elective *vs.* urgent), type and duration of the procedure, type of anaesthesia (regional *vs.* general), hyperglycaemia, dehydration, blood loss, and intraoperative complications such as heart rate alterations or arrythmia.^5^

In general surgery, postoperative strokes are reported to occur in 0.08 to 0.7% of patients^1^ but this is influenced by the type and complexity of the procedure. Major intra-abdominal procedures, such as abdominal exploration, colectomy, and hepatobiliary procedures, carry an increased risk.^4^ This is due to the potential for low perfusion pressure in the brain. Areas particularly vulnerable to hypoperfusion are nonanastomosing arterial vessels (*i.e.* watershed areas). They are critically dependent on adequate perfusion pressure owing to poor collateral circulation. If a watershed area becomes inadequately perfused below a critical threshold, ischaemia and eventual infarction occur. This is the hallmark of haemodynamic stroke.^1^ One could, therefore, presume that hypotension, especially critically low blood pressure, was the cause of the oculomotor stroke.

However, the exact role of hypotension in the aetiology of peri-operative stroke remains unclear. This is because intra-operative hypotension remains a poorly defined term. Depending on the definition used, hypotension can occur in 5 to 99% of patients undergoing surgery.^1^ This makes it difficult to find clear evidence supporting the relationship between hypotension and peri-operative stroke. Of the many studies that investigated this relationship, only a mean blood pressure that decreased more than 30% from baseline blood pressure was associated with the occurrence of postoperative stroke.^6^ The size of the effect from this study was not as large as suggested; therefore, a true cause-and-effect relationship could not be established.

Despite this contention, it is recognised that an unusually low blood pressure for a prolonged period will eventually result in neurological injury. Therefore, hypotension can injure neural tissue directly. It can also act as a secondary factor by compounding the effects of a thrombus or embolus.^1^

In recognising the risk of peri-operative stroke, strategies and methods have been suggested by previous authors to avoid this outcome. This includes avoiding periods of hypotension and maintaining with blood pressure within 20% of baseline for patients at risk of postoperative stroke.^4^ Other notable suggestions include delaying elective surgery for at least 3 months and, if possible, up to 9 months after an ischaemic acute stroke.^4^

Prompt and thorough review of patients postoperatively is another important consideration. Surgical patients are already predisposed to a higher risk of stroke because of the associated pro-thrombotic state. This conveys a greater need for neurological evaluation in the postoperative period, particularly following prolonged periods of hypotension. However, formal neurological observations are generally not mandated in the recovery area or on surgical wards. This potentially prolongs the time before a stroke can be identified. The recognition of acute ischaemic strokes postoperatively also remains a challenge because of anaesthetic factors such as intubation, use of sedation and/or regional anaesthesia being used.

Given that acute ischaemic strokes are time critical events, it is essential that neurological impairments are identified quickly and referred to the appropriate team. This is so that intra-arterial treatment (intra-arterial thrombolysis, mechanical clot retrieval, or both) can be implemented if large vessel occlusion is suspected. Use of assessment tools to identify strokes may, therefore, be of benefit in these circumstances.

For example, the recognition of stroke in the recovery room (ROSIER) tool enables medical and nursing staff to differentiate patients with stroke and stroke mimics. From this case study, the patient scored one point (+1 for asymmetrical facial weakness due to complete right sided ptosis) suggesting a stroke is likely. This may have encouraged earlier detection and therefore swifter response to the oculomotor palsy. Use of tools in the postoperative period may, therefore, prove beneficial in identifying strokes and improving morbidity and mortality in this population.

Fortunately, the prognosis for microvascular ischaemic third nerve palsies is generally good, with improvement developing over several weeks and full resolution occurring within 3 months.^2^ Ptosis usually improves first, and patients often become diplopic again, before complete resolution. If third nerve palsy does not resolve after 3 months, it is recommended that other diagnoses be considered. Patients with residual deficits can consider prisms or strabismus surgery after 6 months of stability.

## Conclusion

The contributing factors of hypertension and diabetes, in combination with prolonged hypotension likely resulted in microvascular ischaemia that produced the oculomotor palsy. Therefore, close attention should be paid to intra-operative blood pressure to avoid hypoperfusion, with key patient risk factors identified and acted upon preoperatively. Close neurological examinations in the peri-operative period may also encourage earlier detection of any postoperative motor deficit and would be of benefit following prolonged periods of intra-operative hypotension. The use of assessment tools (i.e. ROSIER) in the postoperative period could assist in enabling detection, thereby improving morbidity and mortality in the peri-operative stroke population.
